# A Rare Case of Lipomatous Variant Mammary-Type Myofibroblastoma of the Lateral Tongue

**DOI:** 10.1155/2023/1519293

**Published:** 2023-08-26

**Authors:** Matthew Spalti, Firoozeh Samim, Livia Florianova

**Affiliations:** ^1^Faculty of Dentistry, McGill University, 2001 McGill College Avenue, RM 502, Montreal, QC H3A 1G1, Canada; ^2^Department of Pathology, Jewish General Hospital, 3755 Chemin de la Côte Ste Catherine, G-120, Montreal, QC H3T 1E2, Canada

## Abstract

Mammary-type myofibroblastoma (MTMF) is an unusual and rare benign tumor that typically presents in older men or post-menopausal females. A 53-year-old female presented with a 6-month history of an asymptomatic pink/white submucosal nodule involving the left lateral tongue. Clinical examination showed a 5 mm × 5 mm × 5 mm firm submucosal nodule with intact overlying mucosa. Differential diagnoses focused on benign nodular connective tissue tumors. An excisional biopsy was performed and submitted for histopathological examination. The patient underwent conservative local excision. Histopathology and immunocytochemistry revealed a lipomatous variant of MTMF. Hematoxylin and eosin sections revealed an unencapsulated soft tissue lesion with a lobular growth pattern. The neoplasm was biphasic, comprising adipose tissue and cellular fibrous components. By immunohistochemistry, tumor cells were positive for desmin, estrogen receptor, and CD34. In summary, we presented an unusual case of a lipomatous variant of myofibroblastoma on the tongue. MTMF rarely occurs in the head and neck and its accurate diagnosis necessitates awareness of its histomorphological spectrum and application of appropriate immunohistochemical stains.

## 1. Introduction

Mammary-type myofibroblastoma (MTMF) is an unusual benign mesenchymal tumor first described by Wargotz et al. in 1987 as a benign neoplasm of the breast contained in a pseudocapsule composed of spindle cells and abundant collagen [1]. It has since been reported at various extramammary sites. It usually occurs along the embryonic milk line, which extends from the axilla to the medial groin [2, 3]. Howitt and Fletcher examined a series of 143 cases of MTMF and reported extramammary sites and their prevalence, including the inguinal/groin region (65; 45%), breast (15; 10%), chest wall/axilla (7; 5%), trunk (17; 12%), lower (18; 13%) and upper (2; 1%) extremities, or intra-abdominal/retroperitoneal (14; 10%) [4]. In rare circumstances, MTMF can also arise in the head and neck; infrequent case reports of MTMF involving the tongue and infra-auricular region have been reported [5]. Clinically, it is characterized by a slowly growing painless mass that typically presents in older men or post-menopausal females. The diagnosis is based on a constellation of histological and immunohistochemical findings, combined with an appropriate clinical presentation. Literature reports of MTMF of the head and neck are rare, with only a handful of cases described (Table 1). The current case report describes a MTMF of the tongue, which as far as we know, is only the second case reported in the literature thus far.

## 2. Case Presentation

A 53-year-old woman was evaluated for an asymptomatic 5 mm × 5 mm × 5 mm pink/white submucosal nodule involving the left lateral border of the tongue. The overlying mucosa was intact. The lesion had first been noticed by the patient six months before consulting her dentist, who referred her to our clinic for further evaluation.

**Figure 1:**
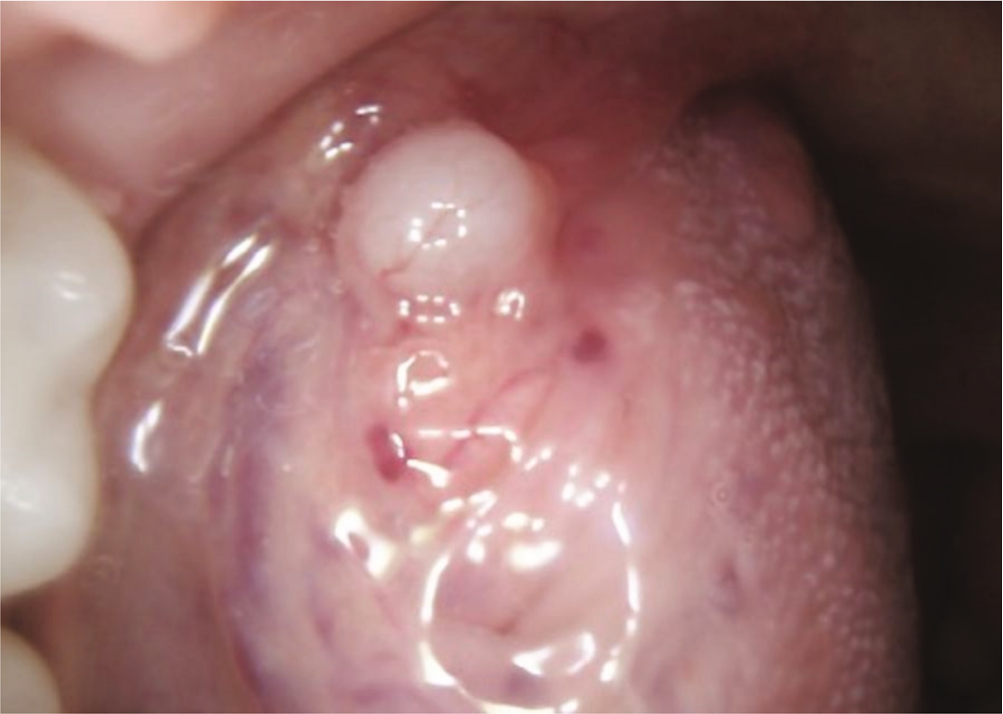
A 5 mm × 5 mm × 5 mm asymptomatic white, pink submucosal nodule involving the left lateral border of the tongue.

The patient's past medical history was only remarkable for hypothyroidism and gastroesophageal reflux disease. Her current medications were Synthroid and occasional pantoprazole. She denied any allergy or history of smoking and consumed alcohol socially (2–3 glasses of wine/week). Her family history was unremarkable. Concerning her tongue lesion, she denied any history of trauma or parafunctional habits, such as grinding or clenching, nor did she notice any other similar lesions at other body sites. There was also no history of gastrointestinal or dermatological issues. Clinical examination of the tongue lesion showed a 5 mm × 5 mm × 5 mm firm submucosal nodule with intact overlying mucosa (Figure 1). Based on the patient's history and clinical examination, an excisional biopsy was performed and submitted for histopathological examination.

## 3. Differential Diagnosis and Discussion

Based on the clinical presentation and stability of the lesion, differential diagnoses focused on benign nodular connective tissue tumors that are more frequently known to affect the tongue. These include fibroma, neurofibroma, hemangioma, schwannoma, granular cell tumor (GCT), leiomyoma, rhabdomyoma, and lipoma. A possible submucosal mucocele was also considered. Nevertheless, a malignant process could not be entirely excluded.

Irritation fibroma and giant cell fibroma were most suspected initially. Irritation fibroma, reactive proliferation of submucosal fibrous tissue, is the most common soft tissue lesion of the oral cavity. It presents as a smooth-surfaced pink nodule similar in colour to the adjacent mucosa, but can often become superficially ulcerated due to localized trauma or chronic irritation [12]. Most fibromas are sessile and can also occasionally be pedunculated. The borders of the lesion are usually well-defined, and it typically grows slowly, rarely exceeding 1.5 cm in diameter. The most common location for irritation fibroma is the buccal mucosa along the bite line; the tongue, labial mucosa, and gingiva are also common sites. This lesion most commonly presents in the fourth to sixth decade of life and the male-to-female ratio is 1 : 2 [13, 14]. Our patient denied any history of trauma, and the lesion was mostly submucosal, which rendered the diagnosis less likely.

Giant cell fibroma presents as an asymptomatic sessile or pedunculated nodule, typically less than 1 cm in diameter. Compared with irritation fibroma, giant cell fibroma typically occurs at a younger age. Furthermore, around 55% of cases occur on the gingiva, with the mandibular gingiva affected twice as often as the maxillary gingiva [13, 14]. The tongue and palate can also be affected. Considering the lesion's firm consistency and lack of colour change relative to the surrounding mucosa, the diagnosis of giant cell fibroma remained a possibility.

In the same category of nodular mesenchymal lesions, lipoma is the most common benign neoplasm. However, lipoma of the tongue represents only 0.3% of tongue tumors [15]. Clinically, lipoma is characterized by a slow-growing, asymptomatic, soft, smooth-surfaced nodular mass that can be sessile or pedunculated. The lesion is typically asymptomatic and less than 3 cm in size but can occasionally be larger. Visually, a subtle yellow hue can often be detected clinically. The most commonly affected intraoral sites are the buccal and vestibular mucosa. Less commonly, lipomas can affect the tongue, floor of the mouth, and lips. Lipomas are mostly found in patients 40 years or older, with no male-to-female predilection. Given our lesion was firm to palpation and pink, the diagnosis of lipoma was less probable.

Considering the presentation as a submucosal swelling, the diagnosis of a mucocele was also reflected in the differential diagnosis. Mucocele is a common lesion of the oral mucosa that results from the rupture of a salivary gland duct and spillage of mucin into the surrounding soft tissues, most often following trauma. Clinically, a mucocele presents as a dome-shaped mucosal swelling ranging from 1 to 2 mm to several centimetres in diameter, occurring primarily in children and young adults. The lesion itself is typically fluctuant but can be firm to palpation. The most commonly affected site is the lower lip, typically lateral to the midline although less commonly, mucoceles can be found in the floor of the mouth, anterior ventral tongue, buccal mucosa, and retromolar pad. Seeing as a mucocele must develop in the area of the oral cavity with minor salivary glands, whereas also considering the firm consistency of our lesion, the diagnosis of mucocele was eliminated from the differential diagnosis.

A neurofibroma and a neurilemmoma (also known as a schwannoma) belong to the category of benign peripheral nerve sheath tumors and can present clinically as a submucosal nodule of the tongue. Neurofibroma is the most common type of peripheral nerve neoplasm and occurs as a reactive soft tissue enlargement of Schwann cells and perineural fibroblasts. It is typically solitary but can be present as numerous lesions in neurofibromatosis. Similarly, a schwannoma is a benign neural neoplasm of Schwann cell origin and typically occurs in association with a nerve trunk. Clinically, both neurofibroma and neurilemmoma present as soft, slow-growing, and asymptomatic masses of varying sizes, ranging in size from a few millimetres to centimetres in diameter. When located intraorally, both lesions are most commonly identified on the tongue, mainly the dorsal and ventral surfaces, and disproportionately affect young to middle-aged adults with a slight female predilection. Occasionally, both neurofibroma and schwannoma can occur centrally in the bone and produce bony expansion. In neurofibroma, this typically occurs in the area of the posterior mandible, whereas in the case of schwannoma, the tumor can occur almost anywhere in the oral cavity. In both instances, the central form of the tumor may produce a well-demarcated or poorly defined unilocular or multilocular radiolucency [16, 17].

A traumatic neuroma is a benign reactive proliferation of nervous tissue occurring in response to injury to a nerve bundle. When repair is interrupted, the proximal area of the nerve supports the disorganized proliferation of Schwann cells leading to a tumor-like mass neuroma. Traumatic neuromas are classically smooth-surfaced, non-ulcerated, and sometimes painful nodules most commonly observed in the area of the mental foramen, tongue, and lower lip. A history of trauma can often be elicited or a recent history of invasive surgical procedures in the affected area. Traumatic neuromas can develop at any age but are most commonly diagnosed in middle-aged adults with a slight predilection for women.

Considering the clinical presentation of our lesion, this category of lesions was considered the most likely diagnosis.

Similar in origin to neurofibroma and neurilemmoma, GCT is a benign soft tissue neoplasm of Schwann cell origin with a predilection for the oral cavity, and more specifically the tongue. Clinically, GCT presents as a solitary, asymptomatic, sessile nodule of less than 2 cm in diameter that is covered by normal pink-coloured mucosa, although it may sometimes appear yellow. GCTs are most often identified in individuals in their fourth to sixth decade of life and the lesion holds a 2 : 1 predilection for females [18, 19]. They are most commonly identified on the dorsum of the tongue. Considering the clinical presentation of our lesion, GCT was considered to be the most probable diagnosis.

Pyogenic granuloma (PG) is a vascular growth of granulation-like tissue that occurs as a response to local irritation or trauma. This lesion is typically smooth or lobulated, pedunculated, and asymptomatic, and the surface is characteristically ulcerated and ranges in colour (pink, red, and purple) depending on the age of the lesion. The size varies from a few millimetres to several centimetres in diameter. Oral PG demonstrates a significant predilection for the maxillary gingiva; however, it can also exist on the lips, tongue, and buccal mucosa in rarer circumstances. In extra-gingival sites, a history of trauma can often be elucidated. PG is most common in children and young adults and has a predilection for females, especially in pregnant women. Our patient denied any history of trauma and our clinical examinations failed to show any erythema or bleeding from the lesion site. As a result, the diagnosis of PG was less probable.

For comprehensiveness, other mesenchymal pathologies, such as hemangioma, leiomyoma, and rhabdomyoma, were also considered in our differential.

Hemangioma occurs as a result of excessive proliferation of endothelial cells lining blood vessels and remains the most common benign tumor of infancy, occurring in 4–5% of 1-year-old children. Clinically, superficial hemangiomas are firm and rubbery to palpation, slightly raised, and bright red due to their blood content. These lesions are typically noted at birth and occur more frequently in whites and show a 3 : 1 female predilection [20].

Leiomyoma is a benign neoplasm of smooth muscle and is extremely rare in the oral cavity. When intraoral, leiomyomas tend to localize on the tongue, lips, cheek, and palate. Extremely rarely, leiomyoma can present intraosseous as a unilocular radiolucency of the jaw. Clinically, this lesion presents as a solitary mucosal nodule of usually less than 2 cm in diameter, asymptomatic, slow-growing, firm, and rubbery on palpation [21].

Rhabdomyoma is an exceptionally rare benign neoplasm of skeletal muscle. Clinically, rhabdomyoma presents most commonly as an asymptomatic, well-delineated, submucosal mass with normal overlying mucosa that can grow to several centimetres before its discovery. When observed intraorally, lesions are typically found in the floor of the mouth, soft palate, and base of the tongue. Rhabdomyoma is most commonly seen in middle to old-age individuals with a slight predilection for men.

Although the clinical presentation of our case suggested benign pathology, ruling out a malignant process is critical. Of particular importance is squamous cell carcinoma (SCC), which constitutes more than 90% of oral malignancies. The development of SCC is related to both intrinsic and extrinsic factors and can often be preceded by a precancerous lesion, presenting in the form of leukoplakia or erythroplakia. SCC has varied clinical presentations, including exophytic or endophytic lesions, leukoplakia, erythroplakia, and erythroleukoplakia. An exophytic lesion typically has a surface that can be irregular, fungating, papillary, or verruciform with colour that varies from normal to white or red, dependent on the quantity of keratin and vascularity in the lesion. The surface is often ulcerated, and the tumor feels hard on palpation. SCC typically presents in older men who have been aware of the oral lesion for months before seeking professional help, mostly because the early growth phase of the lesion is asymptomatic. SCC was considered less likely in this case given our lesion presented submucosally and lacked epithelial changes. However, the possibility of metastasis or submucosal invasion from adjacent tissues could not be ruled out.

None of the lesions discussed here as differential diagnoses cannot be ruled out clinically. Hence, an excisional biopsy specimen under local anaesthesia was taken and sent for histopathological examination.

## 4. Microscopical and Pathological Findings

**Figure 2:**
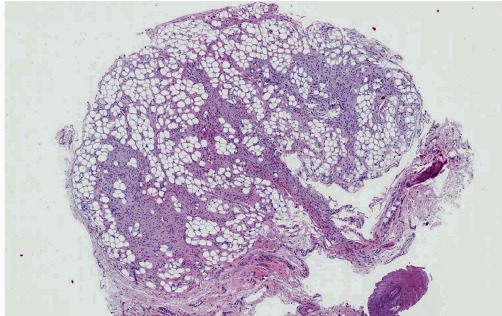
The low-power histological view shows an unencapsulated lobulated biphasic lesion containing adipose tissue and cellular fibrous components.

Macroscopically, the lesion consisted of a nodular fragment measuring 0.3 cm × 0.3 cm × 0.3 cm. Hematoxylin and eosin histological sections revealed an unencapsulated soft tissue lesion with a lobular growth pattern (Figure 2). The neoplasm was biphasic, comprising adipose tissue and cellular fibrous components.

**Figure 3:**
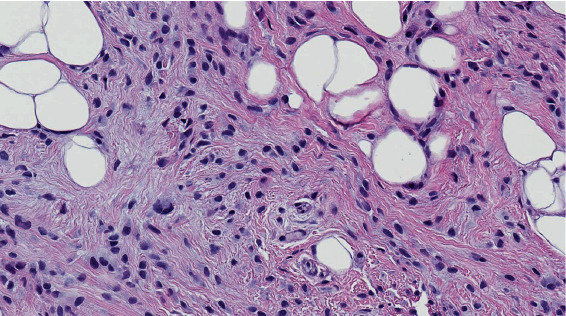
Adipocytes are closely interlaced by fibrous areas populated by loosely packed cells with bland nuclei (some binucleated), on a background of collagen fibers.

Adipocytes were uniform in size and shape and lacked nuclear pleomorphism (Figure 3). This lipomatous component was closely interlaced by irregular fibrous strands of variable thickness, focally forming areas with seemingly entrapped adipocytes. The fibrous component was populated by bland-appearing cells, loosely packed in short haphazardly intersecting fascicles on a background of mostly delicate collagen fibers.

**Figure 4:**
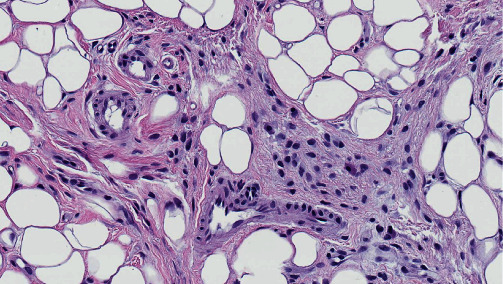
Intralesional vessels are small- to medium-sized with hyalinized walls. Occasional mast cells are scattered among tumor cells.

These cells demonstrated slightly differing morphologies individually, with variable amounts of amphophilic cytoplasm and indistinct cell borders (Figure 4). Most tumor nuclei were round to oval, however, spindle-shaped nuclei were also observed. Occasional cells were binucleated. However, all nuclei were bland, with no or only an indistinct nucleolus. There were no identifiable mitotic figures and no evidence of necrosis. Occasional mast cells were scattered within the lesion among tumor cells. Vessels within the neoplasm were small to medium-sized with hyalinized walls.

**Figure 5:**
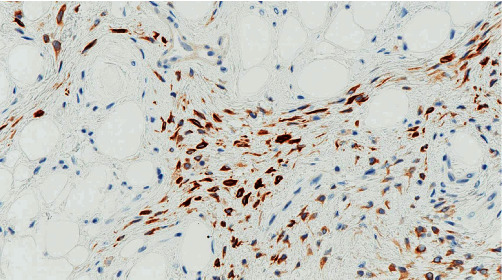
The cytoplasm of myofibroblastoma cells is immmunoreactive for desmin.

**Figure 6:**
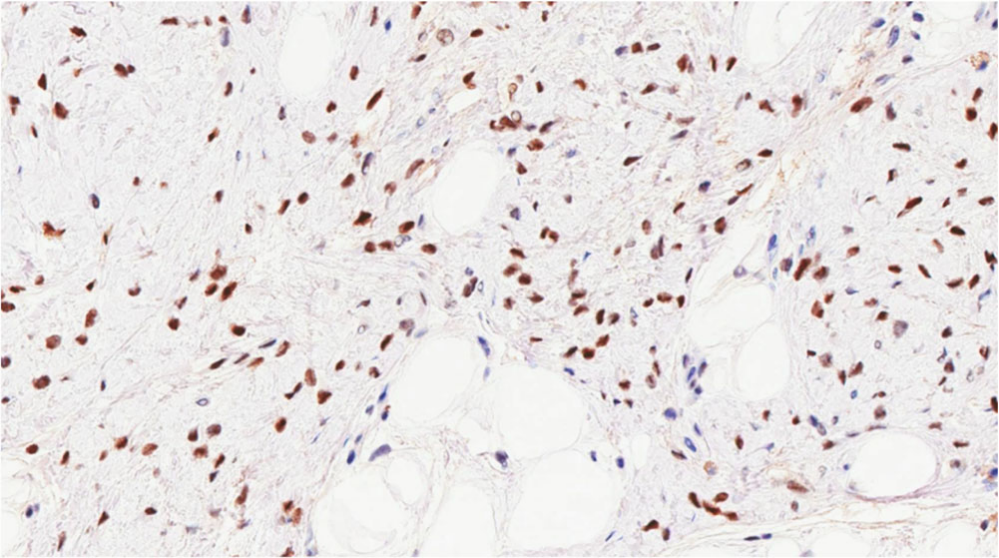
The estrogen receptor immunostain shows nuclear expression in tumor cells, a helpful diagnostic element.

**Figure 7:**
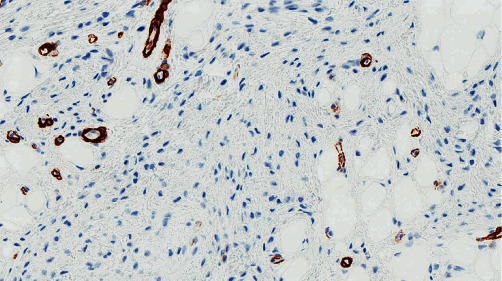
Myofibroblastoma cells are negative for smooth muscle actin; background vessels serve as a positive control.

**Figure 8:**
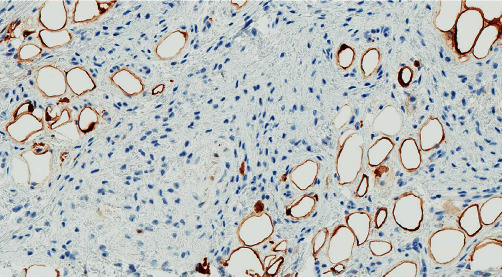
The S100 immunostain highlights intralesional adipocytes but does not show expression in myofibroblastoma cells.

By immunohistochemistry, tumor cells of interest (within the fibrous component) were positive for desmin, estrogen receptor, and CD34 (focal); they were negative for cytokeratin AE1/AE3, SOX-10 (mast cells are immunoreactive), P63, smooth muscle actin, S100 (adipose tissue is immunoreactive), PR, MDM-2, ERG, and HHV-8 (Figures 5, 6, 7, and 8).

Together, the histological and immunohistochemical features were consistent with the diagnosis of a lipomatous variant of myofibroblastoma.

## 5. Management

Accurate diagnosis of MTMF as a benign condition is of extreme importance to prevent radical surgical intervention. There is no evidence of any significant recurrence risk for MTMF, even in the presence of positive resection margins [4, 22]. The patient underwent a conservative local excision under local anaesthesia and had an uneventful postoperative course.

## 6. Conclusion

In summary, we presented an unusual case of a lipomatous variant of myofibroblastoma of the tongue, a benign soft tissue lesion. Myofibroblastoma rarely occurs in the head and neck and the accurate diagnosis of this benign lesion necessitates awareness of its histomorphological spectrum and application of appropriate immunohistochemical stains to confirm the diagnosis. We recommend the inclusion of MTMF in the clinical differential diagnosis of soft tissue tumors of the oral cavity.

## Figures and Tables

**Table 1:** Related cases of myofibroblastoma of the head and neck reported in the literature.

Authors	Type	Age (years)/sex	Location	Size/presentation
Hajeri et al. [6]	Myofibroblastoma	3/M	Right mandible	13 cm × 11 cm mass in the right mandible that extended inferiorly to the neck and posteriorly to the angle of the mandible. The overlying skin was normal. The mass was firm, indurated, and fixed.
Sahin et al. [7]	Myofibroblastoma	77/M	Left tongue border	2 cm × 1.5 cm, non-ulcerated submucosal nodule on the left border of the oral tongue.
Hox et al. [8]	Myofibroblastoma	45/M	Behind the mandibular angle, under the right pinna	6 cm × 6 cm well-circumscribed, mobile, and soft subcutaneous mass behind the mandibular angle.
Luk et al. [9]	Mammary-type myofibroblastoma	58/F	Left buccal mucosa	2 cm × 2 cm mobile, well-circumscribed, and yellowish submucosal lesion with a rubbery consistency under the left buccal mucosa, posterior to the oral commissure.
Kuyumcu et al. [10]	Mammary-type myofibroblastoma	55/F	Floor of the mouth	3.2 cm × 5 cm × 4.2 cm mass situated between the genioglossus and mylohyoid muscles at the floor of the mouth
Hamada et al. [11]	Mammary-type myofibroblastoma	65/F	Left lateral tongue	17 mm × 10 mm × 10 mm solitary, circumscribed, elastic hard, non-tender nodule on the left lateral aspect of the tongue.
